# Utilization of routine health data and its determinants among healthcare workers in public health facilities of harari region, eastern Ethiopia

**DOI:** 10.1186/s12913-024-10834-2

**Published:** 2024-03-19

**Authors:** Gudeta Ayele, Admas Abera, Angefa Ayele, Daniel Gudina, Dawit Firdisa

**Affiliations:** 1 School of Public Health, College of Health and Medical Sciences, Haramaya University, Harar, P.O.BOX, 235, Ethiopia; 2 School of Public Health, Institution of Health, Bule Hora University, Bule Hora, Ethiopia; 3 Médecins Sans Frontières (MSF), East Ethiopia Office, Jigjiga, Ethiopia

**Keywords:** Routine utilization of health data, Healthcare workers, Public health facility, Harari region

## Abstract

**Background:**

Routine health information is the pillar of the planning and management of health services and plays a vital role in effective and efficient health service delivery, decision making, and program improvement. Little is known about evidence-based actions to successively advance the use of information for decision making. Therefore, this study aimed to assess the level and determinants of routine health data utilization among health workers in public health facilities in the Harari region, Ethiopia.

**Methods:**

An institutional-based cross-sectional study design was used from June 1 to July 31, 2020. A total of 410 health care providers from two hospitals and five health centers were selected using a simple random sampling technique. Data were collected through a structured questionnaire complemented by an observational checklist. The collected data were thoroughly checked, coding, and entered into Epi-data version 4.6 before being transferred to Stata version 14 for analysis. Frequency and cross-tabulations were performed. To measure factors associated with routine use of health data, bivariate and multivariate logistic regression analyzes were performed. The odds ratio with a 95% CI was calculated, and then a *p*-value of less than 0.05 was considered significant.

**Result:**

The general utilization of routine health data was 65.6%. The use of routine health data was significantly associated with healthcare workers who had a positive attitude towards data [AOR = 4 (2.3–6.9)], received training [AOR = 2.1 (1.3–3.6)], had supportive supervision [AOR = 3.6 (2.1–6.2)], received regular feedback [AOR = 2.9 (1.7–5.0)] and perceived a culture of information use [AOR = 2.5 (1.3–4.6)].

**Conclusions:**

Sixty percent of health professionals had used routine health data utilization. Training, supervision, feedback, and the perceived culture of information were independently associated with the use of routine health data utilization. Therefore, it is critical to focus on improving data utilization practices by addressing factors that influence the use of routine health data.

## Introduction

A health information system (HIS) is a system that integrates data collection, processing, reporting, and use of the information necessary to improve the effectiveness and efficiency of health services through better management at all levels of the health system [1, 2]. Data from public, private and community health facilities and health organizations produced at systematic intervals encompass routine health information systems (RHIS) [1, 2]. These data provide an image of health status, health services, and health resources. The main purpose of a RHIS is to generate quality information that stakeholders in the health system can use to make evidence-based decisions [1, 2]. It is also the pillar for planning and managing health services at various levels of the health system, as it can play a vital role in efficient and efficient service delivery, decision making, and the improvement of programs [1, 2].

Data utilization is one of the essential components of HIS; it is the analysis, synthesis, and review of data as part of decision-making [3, 4]. Accurate, complete, and timely health information is used to identify strengths and gaps in the health system’s functions and services and, accordingly, to take actions that improve performance [3, 4]. This will be achieved by collecting, processing, and analyzing a series of performance indicators captured mainly through RHIS [3, 4]. In that, it helps managers and health professionals deliver effective clinical management, disease prioritization, planning, drug executives, and monitoring services [5–7].

There is a growing global awareness of the importance of using routine health data for decision-making [3, 7]. Many countries have taken steps to improve their routine health information performance by building capacity, investing in data sources, and leveraging the digital revolution [6, 8, 9]. Despite all these efforts, many developing countries’ RHIS are unable to provide the necessary information [2, 10]. Data generated at the peripheral level often goes underutilized, remaining confined to reports and shelves [11–13]. Weak organizational culture, limited resources, and inadequate infrastructure allocated for RHIS further exacerbate the issue [11–13]. In many developing countries, data producers lack the skills to analyze and interpret data, leading to poor problem identification, resource allocation, and planning [2, 3, 13]. This, in turn, resulted in the failure of many health programs [4, 14]. In Africa, the utilization by health care providers of routine health data is notably low, ranging from 10 to 65% [4, 15–17].

In Ethiopia, despite notable improvements in RHIS collections, reporting and dissemination, the use of routine health information for decision-making remains low [4, 15–17]. Poor data quality, poor access to data, lack of capacity of health managers and providers in core competencies for data use, centralization and fragmentation of health information systems, and poor identification of information needs remain the main barriers in the country to translating data into action [4, 15–17]. A recent finding showed that only 45.8% of healthcare workers adequately utilize data produced in health facilities [18].

Taking into account the above facts, in Ethiopia, the use of local data has been a priority and essential in the transformation process of transforming the health sector [19]. The country has been intensely dedicated to reinforcing its national HIS through different actions. The Federal Ministry of Health (FMOH) has implemented the information revolution, aimed at bringing about a fundamental attitudinal and cultural change with respect to the practical use of data [20, 21].

Several studies have stated that the core determinants of routine health information utilization are technical, behavioral, and organizational factors [22–24]. However, little is known about evidence-based actions to successively progress the use of information for decision making around improving the quality, effectiveness, and efficiency of health service delivery. Therefore, this study aimed to assess routine use of health information and associated factors among health professionals working in public health facilities in the Harari region, eastern Ethiopia. This will have potential to drive positive change in healthcare delivery, data management practices, decision-making processes, and overall health system performance in the Harari Region, and in other healthcare facilities and regions across Ethiopia.

## Materials and methods

### Study design and setting

An institutional-based cross-sectional study design was conducted in 410 healthcare workers from 1 June to 30 July 2020, in the Harari region of eastern Ethiopia. The Harari region is located 515 km from Addis Ababa to the east. There are 12 public health facilities and a total of 1143 healthcare workers have worked in public health facilities in the region. All public health facilities in the region have implemented the DHIS2 system for the collection and reporting of routine data since 2018.

### Study population and sample approach

Study populations were healthcare workers in selected public health facilities in the Harari region of eastern Ethiopia. Healthcare workers who have worked for at least six months in selected public health facilities and are willing to participate in the study were included in the study.

The sample size was determined using both single and double population proportion formulas. The following assumptions were used to calculate the sample size for the first objective: 95% confidence level (1.96), 5% error margin, and proportion (P) of routine use of health data were 45.8% from a similar study conducted in Northern Ethiopia [18]. Accordingly, the sample size was calculated using the formula: n = (z (α/2))^2^ p(1-p)/d2, and after adding 10% nonresponse rate, the calculated sample size was 420. The sample size for the second objective was determined using a double population proportion using Epi Info 7 statistical packages with a 95% CI, a power of 80%, and a 1:1 ratio of exposed to nonexposed. However, the final sample size for the study was the largest sample calculated from a single population proportion.

A simple random sampling technique was used to select the study participants. Of the 12 public health facilities (10 health centers and two hospitals), two hospitals and five health centers were selected using the lottery method. After proportional allocation of samples to each facility, 420 participants were selected using simple random sampling.

### Data collection and quality control

Data were collected using a pre-tested structured questionnaire adapted from the Performance of routine information system management (PRISM) framework tools [25]. The questionnaire contains sociodemographic, level of utilization of routine health data, technical, behavioral, and organizational-related questions. The questionnaire was prepared and administered in English. Additionally, a facility observation checklist was used to assess RHIS-related resources. To maintain data quality, two days of training were given to data collectors and supervisors on the content of the questionnaire and the objective of the study. A pretest was conducted outside the study area on about 5% of the sample size. Daily and strict supervision was provided by supervisors and investigators. The collected data were checked for inconsistency and completeness of entry. Finally, double data entry was performed by two data clerks and cross-validated.

### Operational definition

#### Level routine health data utilization

It was defined as the use of routine health data for eight dimensions (for treating patients, disease prioritization, drug acquisition, day-to-day monitoring of health service activities, checking data quality, planning, and performance evaluation). Then, it was measured using items on a 5-point Likert scale (1 denoting never, 2 seldom, 3 sometimes, 4 often and 5 always). Finally, the participant who scored above the mean score of the healthcare worker was considered to have ‘a good level of data utilization’ or vice versa.

#### Attitude

The degree to which the respondent feels or perceives the usefulness of data use and collection, their responsibility, and the burden of data collection. It was measured using 5-point Likert scale measures ranging from ‘strongly disagree’ to ‘strongly agree’, and the median score was used to label healthcare workers as having a favorable attitude if they scored above the median score for an otherwise unfavorable attitude.

#### Culture of information use

the degree to which healthcare workers perceived the presence of committed managers to seek feedback on affected staff emphasizes data quality, the presence of PMT and use of RHIS data, and incentives for good performance. It was measured by 5-point Likert scale measures, ranging from ‘strongly disagree’ to ‘strongly agree’. Finally, the median score of the healthcare worker was used to classify as perceived good promotion of the information culture for those who scored above or equal to the median score, or vice versa.

#### Self-competence

Respondents rate their level of competence to perform RHIS tasks from 0 to 10 (if they can check data quality, calculate percentages or rates, plot trends on the chart, explain the implications of the results of data analysis, and use data to identify performance gaps). The cut in the median score was used to classify the confidence of the healthcare worker as “strong perceived self-competence” for those scoring above the median score or vice versa.

### Data management and analysis

Data were verified for completeness and entered using Epi-data version 4.6, then exported to STATA version 14. Internal consistency was checked for all computed items (with Cronbach’s alpha). 0.83 for perceived culture of information use, 0.89 for perceived self-competence, 0.80 for attitude toward data use, and 0.81 for routine health data. Frequency and cross-tabulations were used to describe the data. Bivariate analysis and multivariate analysis were performed using the backward method. The odds ratio along with the 95% confidence interval (CI) were estimated. The Hosmer-Lemeshow goodness-of-fit test was used to test for model fitness, and a multicollinearity test was carried out using the Variance Inflation Factor (VIF). Finally, variables with a *p*-value < 0.05 in multivariate logistic regression were considered significantly associated factors.

## Results

### Sociodemographic characteristics of study participants

Of the 420 healthcare workers approached, 410 (96%) participated in the study of which the majority (62.9%) were hospital employees. Just over half (54%) of the respondents were male. The mean age was 29.6 (± 6) years, ranging from 20 to 56 years. Most of the respondents had a degree (83%) and worked in hospitals (62.9%). The majority of the respondents (88.3%) were technical staff, while the rest 11.7% were members of the performance monitoring team (PMT). The average monthly salary of the respondents was ETB 4000 (Table 1).

**Table 1:** Socio-demographic characteristics of healthcare workers working at selected public health facilities of Harari Region, Ethiopia, 2020. (*n* = 410)

Variables	Respondents	Frequency	Percent
Age	< 25	48	11.7
	25–30	245	59.8
	> 31	117	28.5
Sex	Female	189	46.1
	Male	221	53.9
Level of education	Diploma	56	13.7
	Degree	340	82.9
	Masters and above	14	3.4
Position	Regular services providers	362	88.3
	Management members/ PMT	48	11.7
Working experience	< 5	234	57.1
	5–9	140	34.1
	>=10	36	8.8
Monthly salary	< 4000 ETB	32	7.8
	4000–7000 ETB	207	50.5
	> 7000 ETB	171	41.7

### Technical characteristics

A total of 235 (57.3%) participants received training on RHIS of which 36% and 43% were on data analysis and information utilization, respectively. Most of the respondents used paper-based data collection formats. Regarding the usability of data collection tools, 63.9% of respondents described/perceived it as user-friendly (Table 2).

**Table 2:** Technical characteristics of healthcare workers working at public health facilities of Harari Region, Ethiopia, 2020 (*n* = 410)

Variables	Respondents	Frequency	Percent
Training on RHIS	Yes	235	57.3
	No	175	42.7
Types of training received	Data managements	15	6.4
	Data analysis	85	36.2
	Data presentation	35	14.9
	Information use	100	42.6
Perceived data collection formats	User-friendly	262	63.9
	Complex	148	36.1
Primary formats/tools at working unit	Paper-based	307	74.9
	Electronic-based	103	25.1

### Behavioral and organizational characteristics

More than half (52.9%) and 58.7% of the participants received supportive supervision and feedback on the performance of the RHIS tasks, respectively. Of the total of the respondents, 159 (38.8%) of them described that there had been weak leadership. Regarding the attitude of the respondents towards RHIS tasks and data use, 36% of the respondents believed that performing RHIS tasks was a tedious (repetitive) action, 51% of the participants did not perceive their role and their responsibility to collect data, while 52.8% of the participants perceived the benefits of using routine health data. In general, 49.5% of the participants have a favorable attitude toward RHIS tasks based on the median score of the healthcare worker (Table 3).

**Table 3:** Behavioural and organizational characteristics of healthcare workers working at public health facilities of Harari Region, Ethiopia, 2020 (*n* = 410)

Behavioral aspects	Respondents	Frequency	Percent
Attitude towards RHIS tasks	Favorable attitude	207	50.5
	Unfavorable attitude	203	49.5
	Total	410	100
Perceived self-competence to performs RHIS task	Strong perceived self-competence	191	46.6
	Weak perceived self-competence	219	53.4
	Total	410	100
Perception on data quality	Good	238	58
	Poor	172	42
	Total	410	100
Supportive supervision on RHIS tasks	Yes	217	52.9
	No	193	47.1
	Total	410	100
Perceived leaderships of facility	Strong	251	61.2
	Weak	159	38.8
	Total	410	100
Perceived culture of information use	Good	229	55.9
	Poor	181	44.1
	Total	410	100
Regular feedback	Yes	240	58.5
	No	170	41.5
	Total	410	100

Regarding perceived data quality, 58% of participants perceived the exitance of good quality. Moreover, just about 53.4% of the respondents had a strong self-competence in performing RHIS tasks. Regarding the culture of information about 229 (55.9%), respondents perceived the presence of a good culture of information use (Table 3).

### Level of utilization of routine health data

In this study, the level of routine health data utilization was measured by computing the values of nine different dimensions of routine health data utilization. Consequently, of the total of the respondents, 269 (65.6%) of the respondents had a good utilization of routine health data, since they scored above the mean value (Table 4).

**Table 4:** Mean score and relative important index for routine health data utilization among healthcare workers in public health facilities of Harari Region, Eastern Ethiopia, 2020 (*n* = 410)

Dimension of RHIS data utilization	Mean score	Relative important index
Treating patients/ clinical services	3.98	0.80
Disease’s prioritization	3.81	0.76
Planning	3.81	0.76
Monitoring performance	3.32	0.66
Data quality checking	3.61	0.72
Health education	3.88	0.78
Drug procurement	3.32	0.66
Dept. performance evaluation	2.88	0.58
Total		
Overall level of routine health data utilization = 65.6%		

### Determinants factors of routine health data utilization

In the bivariate logistic regression analysis, sex, position, training, perceived data collection format, attitude, perceived self-competence, a culture of information use, feedback, supervision, perceived data quality and leadership were factors associated with the utilization of routine health data at a *p*-value of less than 0.25. Consequently, these variables were subjected to a multivariate logistic regression analysis (Table 5).

**Table 5:** Multiple logistic regression and factors associated with utilization of routine health data Harari Region, Ethiopia, 2020 (*n* = 410)

Variable	Categories	RHIS data utilization		COR With 95% CI	AOR With 95% CI
		Good	Poor		
Sex	Male Female	154 115	67 74	1.4 (0.9–2.2) 1	1 1.1 (0.6–1.6)
Staff based on types facility	Hospital Health center	143 115	126 26	1 3.9 (2.4–6.4)	1 2.9 (0.91–5.4)
Position	Service providers PMT members	230 39	132 9	1 2.5 (1.2–5.3)	1 1.8 (0.73–4.8)
Training on RHIS	No Yes	90 179	85 56	1 3.0 (1.9–4.6)	1 2.1 (1.3–3.6) *
Data collection formats	Complex User-friendly	84 185	64 77	1 1.8 (1.2–2.7)	1 1.4 (0.8–2.4)
Attitude towards RHIS	Unfavorable Favorable	97 172	104 35	1 5.3 (3.4–8.4)	1 4 (2.3–6.9) *
Competence to performs RHIS	Weak Strong	121 148	98 43	1 2.8 (1.8–4.2)	1 1.2 (0.7–2.0)
Perceived data quality	Poor quality Good quality	92 177	80 61	1 2.5 (1.6–3.8)	1 1.4 (0.8–2.4)
Supportive supervision	No Yes	100 169	93 48	1 3.8 (2.4–5.9)	1 3.6 (2.1–6.2) *
Regular feedback	No Yes	85 184	85 56	1 3.3 (2.1–5.0)	1 2.9 (1.7–5.0) *
Leadership	Weak Strong	80 189	79 62	1 3.1 (1.9–4.6)	1 1.4 (0.7–2.6)
Culture of information use	Weak Strong	85 184	96 45	1 4.6 (2.9–7.2)	1 2.5 (1.3–4.6) *

*- significant at *p*-value < 0.05

In multivariate analysis, the odds of good utilization of routine health data were almost three times higher among healthcare workers who had worked in the health center compared to hospital staff [AOR = 2.9; 95% CI: (1.6– 5.4)]. Furthermore, the chances of good utilization of routine health data among trained individuals were twice that of those without RHIS training [AOR = 2.1; 95% CI: (1.3– 3.6)]. Respondents with a favorable attitude towards the execution of RHIS tasks were four times more likely to use routine health data compared to their counterparts [AOR = 4.0; 95% CI: (2.3–6.9)]. Furthermore, the oddity of good utilization routine health data was two and half times [AOR = 2.5; 95% CI: (1.4–4.6)] more likely higher among health professionals who perceived the presence of information culture in their facility than their counterparts (Table 5).

## Discussion

This study aimed to identify the level of utilization of routine health data and its determinant factors among healthcare workers. Thus, the study revealed that about two thirds (65.5%) of healthcare workers were good users of routine health data. It is higher than the study 45.8% in Northwest Ethiopia [18], 57.9% in Easter Wallaga [26], 38.4% in Northern Ethiopia [27], in Kenya 48.1% [28], and Uganda 59% [17]. These might be attributed to better implementation of the information revolution, particularly in terms of use of digital health. The other reason could be attributed to the presence of different stakeholders providing technical assistance on RHIS (CBMP project, and others). However, this finding is lower than studies in the north Gondar zone in the Amhara region (78.5%) [29] and the Hadiya zone (69.3%) [30]. This could be due to the difference in the type of facility and the participants and the criteria used to measure data utilization.

Training in basic RHIS tasks was found to be one of the technical determinants of routine use of health data. This factor was repeatedly mentioned as crucial to the utilization of the data by various studies done in different places [13, 18, 31]. This because trained healthcare workers had the potential to compile, analyze, and use data in their day-to-day decision-making [32], these contribute to improved self-competence and motivation of Healthcare workers [12, 13]. Furthermore, participants from health centers were three times more likely to be a good user of routine data compared to participants from hospitals. The finding corresponds to studies conducted in Northern Ethiopia [18, 29]. This might be due to the great attention paid by the government to district health facilities [29].

Furthermore, participants who had regular feedback were 3.6 times more likely to use routine data than their counterparts. This finding corresponds to previous studies conducted in a different place [26, 27, 31]. Healthcare workers better understand the value of data and are motivated to use data if they are provided feedback regularly [13]. Supportive supervision was also the organizational factor that was distinctly associated with routine data utilization. This factor was also mentioned as vital to the use of data utilization by various studies [12, 28, 29]. This could be attributable, since supervision enables healthcare workers to identify the gaps and improve the performance [18].

Perceived culture information use was another major determinant of routine data utilization. This finding is in line with various studies [14, 31, 33]. If an organization actively promotes the value of evidence-based decision making and incentives to collect and use data, the motivation and attitude of data users toward data use is more likely fostered [4, 33]. Furthermore, the attitude of healthcare workers toward RHIS tasks was found to be a determinant of the use of routine health data. These factors have been repeatedly reported by others as the main determinant of data utilization [33]. If healthcare workers do not realize the value of using data or do not appreciate the usefulness of the data they collected and utilized for decision making [32].

## Conclusion

Routine health data utilization was found among two-thirds of healthcare workers and was below the recommended level. Additionally, the type of facility, the training, supervision and feedback, the perceived culture of information and the attitude of the healthcare worker toward the RHIS tasks were independent predictors of routine use of health data. Therefore, it is critical to focus on improving data utilization practices by addressing factors that influence the use of routine health data. By prioritizing these areas, healthcare systems can promote effective data utilization, enhance decision-making, and ultimately improve overall healthcare delivery and outcomes.

## Data Availability

The datasets used during the current study are available from the corresponding author upon reasonable request.
